# Middlesex Hospital

**Published:** 1900-12-08

**Authors:** 


### MIDDLESEX HOSPITAL.

A quarterly general court of governors of the Middlesex Hospital was held on Thursday, November 29th ult., Sir Arthur Watson presiding. The report of the weekly board stated that during the past quarter progress had been made with the various schemes for alterations and extensions now in hand. In consequence of difficulties which arose out of the plans submitted for a new laundry it had been found necessary to abandon in part the scheme adopted last March; and it had now been decided to recommend the construction of a new laundry on a site altogether outside the hospital, a piece of land for which was still being sought. Revised plans were therefore submitted which had been adopted by the Building Committee and approved by the Medical Committee, It was now proposed to retain intact the 26 beds in the old cancer wards and to extend the north-east wing. This extension, besides being desirable in itself, was necessary to provide space for the kitchen offices of the hospital, which will be transferred to the third floor. There will be a complete sanitary block at the end of each floor. The architect had been instructed to proceed with the detailed plans and to obtain estimates for the work. Meanwhile the board had decided, with the concurrence of the Medical Committee, to bring the old cancer wards into immediate use for gynæcological cases, and had accepted an estimate at £87 for the requisite cleaning and painting. The designs for additional nurses' accommodation and the site of the three old houses recently demolished in Cleveland Street had been prepared, but could not be proceeded with until the building extension to the northern wing had been completed. The plans for centralising the boiler and engineering departments would be much simplified by the exclusion of the laundry, and fresh schemes, taking this into account, had been called for. The installation of the new electric coal lift had been completed, but in consequence of unforeseen difficulties necessitating structural alterations, there had been a considerable addition to the estimated expenditure on this lift. The meeting was asked to sanction the payment of the contractor's certified account for this work, amounting to £175, as well as £177 for repairs to soil-pipes incurred after a careful examination of the whole drainage system of the hospital and its adjuncts. Negotiations had been completed with the trustees of the Berners Estate for a lease of 5 Nassau Street, adjoining the new cancer wing, for 93 years from 1905, at £50 per annum.

The tenure of office of chaplain of the Rev. W. G. Deighton expires next month, and the board stated that the qualifications of applicants for the post were being considered. It had been decided that the new appointment should be for two years, the holder to be eligible for re-election for a third year, but that the maximum tenure of the office should be three years. Mr. S. B. Hulke, now in South Africa, had resigned the office of surgical registrar, and Mr. Aslett Baldwin, who had been acting as his substitute, had consented to continue the duties of the office till the end of the year. Dr. J. C. Thorowgood, no longer residing in London, had retired from the Drug Committee. He had rendered valuable services to the hospital over a period of twenty-eight years. In view of the importance of the photographic and radiographic work a skilled photographer had been engaged, to act under the supervision of the registrars. It was recommended that the salary of the Pharmacist, Mr. John Kitchin, be increased by annual increments to a maximum of £250. Subscriptions were invited for the purpose of placing a memorial window in the chapel to the late Mr. Storer Bennett, Consulting Dental Surgeon. The Board had signified their desire cordially to support the steps taken by the Central Hospital Council for London to initiate legislation during the coming session on the lines indicated in the report of the select committee of the House of Commons on the exemption of hospitals from local rates. Among other donations recorded was one from Miss Wicksteed of £1,050 for the endowment of a bed in memory of her sister; while, on the other hand, it was mentioned that the annual subscription of 50 guineas hitherto paid by the Drapers' Company, would, in future, be paid to the Prince of Wales's Fund.

To repay loans from the bankers and for current expenses, the meeting was asked to authorise a sale of stock to realise £8,500.

In the Medical School, the winter session of which was opened by an Introductory Address to the students by Professor Clifford Allbutt, Dr. Walter Essex Wynter had resigned his medical tutorship, having been appointed Lecturer on Practical Medicine. Dr. H. Campbell Thomson had been appointed to succeed him. During the quarter ended September 30th, 1,088 patients were treated in the hospital. In the female cancer wing 31 new patients had been admitted, and there had been 29 deaths. In the out-patient department 13,976 patients were treated, with an average of 2·2 attendances for each patient. 179 had been received into the Convalescent Home during the quarter.

The Chairman briefly moved the adoption of the report which, he said, set forth the ultimate wishes of the Board with regard to the contemplated alterations. The questions, involved had been of considerable difficulty, and the Building Committee had been at great pains to decide what was necessary and desirable to be done. The medical staff had always wished to keep the cancer ward, though it had been decided to take it for the kitchens, and he was glad they had been able to arrange that the kitchens should be elsewhere, though still upstairs. The removal of the laundry was also a very good thing, though the cost of carriage to and fro would, of course, add something to their expenses. As to the relief of hospitals from rates, he hoped all who had influence with members of Parliament would use it to get this carried.

Mr. Ormerod seconded the motion and emphasised the desirability of the removal of the laundry from the hospital from a sanitary point of view and also by reason of the increased space left at their disposal.

The report and its recommendations were carried *nem. con.*; and other regular business having been transacted, the meeting separated after recording votes of thanks to the retiring chaplain and to the chairman for presiding.

